# Child hunger and the protective effects of supplemental nutrition assistance program (SNAP) and alternative food sources among Mexican-origin families in Texas border *colonias*

**DOI:** 10.1186/1471-2431-13-143

**Published:** 2013-09-13

**Authors:** Joseph R Sharkey, Wesley R Dean, Courtney C Nalty

**Affiliations:** 1 Program for Research and Outreach-Engagement on Nutrition and Health Disparities, Texas A&M School of Rural Public Health, MS 1266, College Station, TX 77843-1266, USA

**Keywords:** Childhood hunger, Food assistance programs, Alternative food sources, Immigrant

## Abstract

**Background:**

Nutritional health is essential for children’s growth and development. Many Mexican-origin children who reside in limited-resource *colonias* along the Texas-Mexico border are at increased risk for poor nutrition as a result of household food insecurity. However, little is known about the prevalence of child hunger or its associated factors among children of Mexican immigrants. This study determines the prevalence of child hunger and identifies protective and risk factors associated with it in two Texas border areas.

**Methods:**

This study uses 2009 *Colonia* Household and Community Food Resource Assessment (C-HCFRA) data from 470 mothers who were randomly recruited by *promotora*-researchers. Participants from *colonias* near two small towns in two South Texas counties participated in an in-home community and household assessment. Interviewer-administered surveys collected data in Spanish on sociodemographics, federal food assistance program participation, and food security status. Frequencies and bivariate correlations were examined while a random-effects logistic regression model with backward elimination was used to determine correlates of childhood hunger.

**Results:**

Hunger among children was reported in 51% (*n* = 239) of households in this C-HCFRA sample. Bivariate analyses revealed that hunger status was associated with select maternal characteristics, such as lower educational attainment and Mexican nativity, and household characteristics, including household composition, reliance on friend or neighbor for transportation, food purchase at dollar stores and from neighbors, and participation in school-based nutrition programs. A smaller percentage of households with child hunger participated in school-based nutrition programs (51%) or used alternative food sources, while 131 households were unable to give their child or children a balanced meal during the school year and 145 households during summer months. In the random effects model (RE = small town), increased household composition, full-time unemployment, and participation in the National School Lunch Program were significantly associated with increased odds for child hunger, while participation in Supplemental Nutrition Assistance Program (SNAP) and purchasing food from a neighbor were significantly associated with decreased odds for child hunger.

**Conclusions:**

This study not only emphasizes the alarming rates of child hunger among this sample of Mexican-origin families, but also identifies economic and family factors that increased the odds for child hunger as well as community strategies that reduced the odds. It is unsettling that so many children did not participate in school-based nutrition programs, and that many who participated in federal nutrition assistance programs remained hungry. This study underscores the importance of identifying the presence of child hunger among low-income Mexican-origin children in Texas border *colonias* and increasing access to nutrition-related resources. Hunger-associated health inequities at younger ages among *colonia* residents are likely to persist across the life span and into old age.

## Background

Hunger among children is a serious public health problem in the U.S. and reflects an insufficient quantity and quality of food consumed [1,2]. The Community Childhood Hunger Identification Project (CCHIP) defined hunger “as the mental and physical condition that comes from not eating enough food, due to insufficient economic, family or community resources” [3]. Others have referred to hunger that is due to limited assets as resource-constrained hunger [4]. The uncertainty of adequate food supplies among Hispanics and Mexican-origin U.S. households exceeds national estimates and is more common in households with children than in those that are childless [5,6]. At the same time, nutrition-related health conditions, such as obesity and type 2 diabetes, are more prevalent and dramatically increasing in Mexican-origin children than other racial/ethnic groups [7-9]. Very low food security (formerly labeled food insecurity with hunger) describes disrupted eating patterns and reduced intake because of insufficient resources [10]. Prior reports have classified food insecurity with hunger as present when one or more household members were hungry at least some time during the described time period because they could not afford enough food [11]. Among children of Mexican immigrant parents, very low food security is associated with greater dietary intakes of total calories and percentage of calories from fat and added sugar [12].

Child hunger, as both a poor outcome and risk factor for adverse health and development [6], is a serious challenge facing children [13,14]. A direct link has been established between inadequate food quality and quantity and poor mental and physical health, psychosocial, behavioral, learning, family stress, and academic outcomes [3,15-24]. Mexican American children and children living in immigrant households are at the greatest risk for hunger [6,14,25-27]. Texas is home to the second largest number of foreign-born residents from Mexico and more than 1.8 million children living in food insecure homes (the second largest absolute number of children living in food insecure households in the U.S.), with the greatest density in Texas near the border with Mexico [28,29].

**Figure 1:**
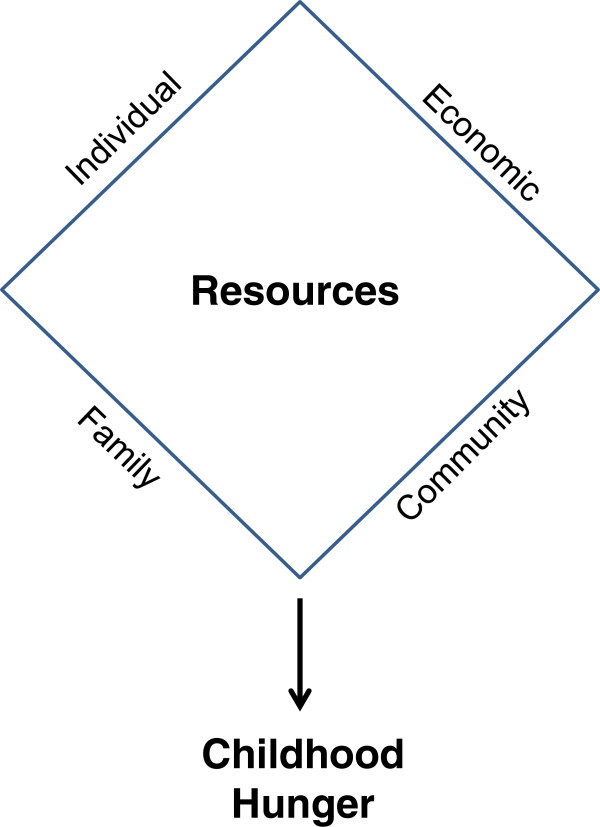
Conceptual model for the influence of resources on childhood hunger.

As depicted in Figure 1, the presence or absence of economic (available financial resources and employment), family (relatives and household composition), community (accessible transportation systems, neighbors, utilization of public nutrition assistance programs, numbers and types of retail food stores, and emergency food programs), and individual resources (education, knowledge, and household features) may serve as protective or risk factors for hunger [13,30]. Although the reduction of child hunger requires an understanding of the determinants of child hunger, there is little known about the protective and risk factors of child hunger among the growing Mexican immigrant population in the U.S., especially residents of the expanding *colonias* or settlements along the Texas-Mexico border. *Colonia* residents share a similar history, language, and socioeconomic status and have profoundly high rates of poverty and food insecurity [27,31,32]. Further, border-region *colonias* can be considered a prototype for the increasing number and geographic dispersion of new destination immigrant communities [31]. This study uses data from the 2009 *Colonia* Household and Community Food Resource Assessment (C-HCFRA) to examine child hunger among 470 Mexican-origin families by (1) determining the prevalence of child hunger and 2) identifying protective and risk factors associated with hunger among children.

## Methods

The 2009 C-HCFRA was conducted in 44 *colonias* in two geographic areas in the Texas border region between September 10, 2009 and September 28, 2009: 19 *colonias* in or near the small town of Progreso in Hidalgo County and 25 *colonias* in or near La Feria in Cameron County. The point prevalence survey was conducted to document food insecurity, food access, and retail food store utilization among 610 Mexican-origin families along the Texas-Mexico border. Details of training, participant eligibility and recruitment, and administration of the Spanish-language in-person survey by highly trained *promotora*-researchers have been previously described in detail [27]. *Promotora*-researchers are community-based health workers, certified as Community Health Workers (C.H.W.) by the State of Texas, Human Subjects certified, and are able to establish trust with respondents [33]. There were two teams of two p*romotora*-researchers who conducted the surveys in the same areas; one team of two worked in the Progreso area and the other team of two worked in the La Feria area. All four p*romotora*-researchers resided in Hidalgo County, but had outreach experience in multiple Lower Rio Grande Valley counties. The survey instrument was specifically developed for this project and all protocols were approved by the Texas A&M Institutional Review Board, and each parent/caregiver provided informed consent. Since the focus of this analysis was child hunger, data were restricted to 470 (77.0%) households with at least one child under the age of 18 years in residence. All surveys were conducted in Spanish.

### Measures

C-HCFRA survey data included individual resources (age, education, and country of birth of parent/caregiver), economic resources (household income and employment status for all adults), family resources (household composition and transportation), and community resources (retail food stores used, participation in nutrition assistance programs, perception of the food environment, and alternative food sources). Nutrition assistance programs included Supplemental Nutrition Assistance Program (SNAP), Special Supplemental Nutrition Program for Women, Infants, and Children (WIC), School Breakfast Program (SBP), and National School Lunch Program (NSLP). Perception of the community (local) food environment was assessed by three items on a four-point Likert scale (strongly agree, agree, disagree, or strongly disagree): 1) little variety in the types of foods that can be purchased; 2) few grocery stores or supermarkets; and 3) high food prices [27]. Binary variables were constructed as strongly agree/agree vs. all others. The utilization of alternative retail food sources included the purchase of food from mobile food vendors, *pulgas* (flea markets), and neighbors or friends [34,35]. Respondents were not asked about their immigration status.

Hunger was measured using two items from the 12-item Radimer/Cornell measure of hunger and food insecurity [36,37]. Adult hunger was defined by responding that the following statement was ‘sometimes true’ or ‘often true’: “I am hungry but don’t eat because I can’t afford enough food.” Child hunger was defined by responding that the following statement was ‘sometimes true’ or ‘often true’: “I know my child(ren) is (are) hungry sometimes, but I just can’t afford more food.” Adult hunger and child hunger were each constructed as binary variables: present (sometimes/often true) vs. absent (not true).

### Analysis

All Spanish-language survey data were recorded in a relational database using double-key entry to minimize data entry error. All statistical analyses were conducted using Stata Statistical Software (Release 12.1, 2011, Stata Corporation, College Station, TX); *p* <0.05 was considered statistically significant. Descriptive statistics were estimated for the total sample and by child hunger status. Bivariate associations between all measures and child hunger (child hunger vs. no child hunger) were calculated using chi square analyses for categorical variables and Student’s *t*-tests for continuous variables. To account for correlation of child hunger by geographical location, a random effects (RE) logistic regression model was used with households nested within small town of residence (Progreso vs. La Feria) [38]. All statistically significant (*p* ≤ 0.10) predictor variables from bivariate analyses were simultaneously entered into the model. Backward elimination sequentially removed non-significant variables in order to obtain the “best” set of independent variables [39]. Model goodness of fit was assessed using Akaike Information Criterion (AIC), which is a measure of fit and complexity [40,41].

## Results

Data included all 470 participants in the 2009 C-HCFRA in the Texas border region who reported that at least one child under the age of 18 years resided in the home. Almost fifty-one percent reported child hunger – their “child(ren) is(are) hungry sometimes, but [parents/caregivers] can’t afford more food.” Sixteen percent of respondents who reported no child hunger indicated adult hunger; and 41.3% (*n* = 194) of households reported no adult or childhood hunger. Descriptive statistics are shown in Table 1 and Table 2. In the Total columns of both tables, the results are displayed for all households with children. The comparison between households without child hunger and households with child hunger are shown in the No Child Hunger and Child Hunger columns in both tables. Respondents who reported child hunger were significantly older, less educated, more likely to be born in Mexico, received less household income, lived in larger households, had a larger number of school-aged children, and more often than not resided in Progreso. Among the 1,126 children who resided in the study households, 53.1% resided in child-hungry households. Unemployment in participant households at the time of the survey was very high in comparison to the concurrent rate of 8.2% unemployment in Texas, with 10% in La Feria, and 11% in Progreso for all households; there were no adults employed full-time in 48% of households, and no adults employed full- or part-time in 18% of households. Compared with households with no child hunger, proportionately more households with child hunger relied on a friend or neighbor for transportation to purchase groceries. In data not shown, the mean number of children under the age of 18 years did not differ by area (2.4 for Progreso vs. 2.3 for La Feria). Children were categorized into three age groups: children under 5 y (WIC-eligible ages), children 5 y (not eligible for WIC or school-based nutrition programs), and children 6–17 y (school-aged children eligible for school-based nutrition programs). In households with at least one child under the age of 5 years (57% of all households), there were more children in La Feria households than Progreso (1.4 vs. 1.3, *p* =0.047). In households with school-age children, the number of school-age children was greater in Progreso households than La Feria (2.2 vs. 1.7, *p* <0.001). Fourteen percent of households had at least one child age 5 years, which could be considered a “donut hole” age; that is, too old for WIC and too young for school-based programs. For this age group, there was no statistically significant difference based on child hunger status or area of residence.

**Table 1:** **Individual, economic, and family resources for the study population and by presence of child hunger (*n*** **= 470)**

	**Total**		**No child hunger**		**Child hunger**	
	**(*n*** **= 470)**		**(*n*** **= 231)**		**(*n*** **= 239)**	
	**% ( *n *)**	**Mean ± SD (Median)**	**% ( *n *)**	**Mean ± SD (Median)**	**% ( *n *)**	**Mean ± SD (Median)**
***Area of Residence***						
La Feria	45.5 (214)		74.9 (173)		17.2 (41)	
Progreso	54.5 (256)		25.1 (58)		82.8 (198)^***^	
***Individual Resources***						
Age, y^a^		36.5 ± 10.9 (35)		35.2 ± 10.3 (34)		37.7 ± 11.3^**^ (36)
Education						
< 7th grade	25.7 (116)		19.7 (44)		31.6 (72)^**^	
Country of birth						
Mexico	68.5 (322)		61.0 (141)		75.7 (181)^***^	
***Economic Resources***						
Household income						
Not know/ refused	24.7 (116)		22.9 (53)		26.4 (63)	
< $500/ month	30.8 (145)		26.0 (60)		35.6 (85)^*^	
$500 - $999/ month	28.9 (136)		27.7 (64)		30.1 (72)	
$1000-$1500/ month	9.4 (44)		13.4 (31)		5.4 (13)^**^	
> $1500/ month	6.2 (29)		10.0 (23)		2.5 (6)^***^	
Employment status						
No adult employed full-time or part-time	17.9 (84)		15.2 (35)		20.5 (49)	
No adult employed full-time	48.3 (227)		46.3 (107)		50.2 (120)	
***Family Resources***						
Female head of household	14.0 (66)		14.7 (34)		13.4 (32)	
Household Composition^b^		4.5 ± 1.6 (4)		4.3 ± 1.7 (4)		4.7 ± 1.5^**^ (5)
1st quartile (2–3)	25.3 (119)		29.0 (67)		21.8 (52)	
2nd quartile (4)	27.2 (128)		32.5 (75)		22.2 (53)^**^	
3rd quartile (5)	27.0 (127)		21.2 (49)		32.6 (78)^**^	
4th quartile (>5)	20.4 (96)		17.3 (40)		23.4 (56)	
Children in household^c^		2.4 ± 1.3 (2)		2.3 ± 1.2 (2)		2.5 ± 1.3 (2)
Child under 5 y^d^		1.4 ± 0.6 (1)		1.3 ± 0.6 (1)		1.4 ± 0.6 (1)
Children 5 y^e^	14.3 (67)		16.9 (39)		11.7 (28)	
Children 6–17 y^f^		1.9 ± 0.9 (2)		1.8 ± 0.8 (2)		2.0 ± 1.0^*^ (2)
Transportation for groceries						
Ride with friend or neighbor	29.1 (137)		17.3 (40)		40.6 (97)^***^	
Car available during day	67.2 (316)		60.2 (139)		74.1 (177)^***^	

^a^Age of adult participant.

^b^Total of adults and children living in the household.

^c^Total of children in the household under the age of 18 y.

^d^At least one child in the household under the age of 5 y (*n* = 268 households).

^e^At least one child age 5 y in households (*n* = 67 households).

^f^At least one child in the household ages 6–17 y (*n* = 359 households).

SD = standard deviation. Comparison between household with no child hunger vs. child hunger: binary variables (cross-tabs with χ^2^ statistic) and continuous variables (Student’s *t*-test). Statistical significance: ^*^p < 0.05, ^**^p < 0.01, ^***^p < 0.001.

**Table 2:** **Community resources by presence of child hunger (*n*** **= 470)**

	**Total**		**No child hunger**		**Child hunger**	
	**(*n*** **= 470)**		**(*n*** **= 231)**		**(*n*** **= 239)**	
	**% ( *n *)**	**Mean ± SD**	**% ( *n *)**	**Mean ± SD**	**% ( *n *)**	**Mean ± SD**
Grocery purchase^a^						
Supermarket	60.4 (284)		70.1 (162)		51.0 (122)^***^	
Supercenter	59.6 (280)		46.7 (108)		72.0 (172)^***^	
Dollar store	24.3 (114)		9.1 (21)		38.9 (93)^***^	
Nutrition assistance programs						
SNAP	63.4 (298)		67.5 (156)		59.4 (142)	
Amount ($)		357.20 ± 198.76		363.29 ± 215.34		350.51 ± 179.32
Days last^b^		20.9 ± 7.7		22.4 ± 7.6		19.2 ± 7.5^***^
<14 days	10.2 (30)		7.8 (12)		12.9 (18)	
14-20 days	23.5 (69)		16.9 (26)		30.7 (43)^**^	
21-30 days	66.3 (195)		75.3 (116)		56.4 (79)^***^	
WIC^c^	70.1 (188)		65.5 (93)		75.4 (95)	
SBP^d^	61.8 (222)		75.0 (123)		50.8 (99)^***^	
NSLP^d^	62.1 (223)		75.0 (123)		51.3 (100)^***^	
Emergency	1.9 (9)		1.7 (4)		2.1 (5)	
Local food environment						
Little variety	92.1 (433)		89.2 (206)		95.0 (227)^*^	
Few grocery stores	93.0 (437)		90.0 (208)		95.8 (229)^**^	
High prices	94.7 (445)		92.2 (213)		97.1 (232)^*^	
Food challenges						
No balance – school year^e^	30.6 (144)		5.6 (13)		54.8 (131)^***^	
No balance - summer^f^	38.9 (183)		16.4 (38)		60.7 (145)^***^	
Alternative food source^g^						
Neighbor/friend	26.2 (123)		36.8 (85)		15.9 (38)^***^	
MFV	31.7 (149)		28.1 (65)		35.1 (84)	
*Pulga*	31.7 (149)		38.5 (89)		25.1 (60)^***^	

^a^Participants could identify more than one store.

^b^Among 294 SNAP recipients who answered this question.

^c^Among 268 households with a child under 5 y.

^d^Among 359 households with school-age children (6–17 y).

^e^Unable to give my child(ren) a balanced meal during the school year because I can’t afford it.

^f^Unable to give my child(ren) a balanced meal during the summer because I can’t afford it.

^g^Participants responded to use of each type of alternative food source.

SNAP = Supplemental Nutrition Assistance Program; WIC = Special Supplemental Nutrition Program for Women, Infants, and Children; SBP = School Breakfast Program; NSLP = National School Lunch Program; Emergency = food bank, food pantry, church; MFV = Mobile Food Vendor; *Pulga* = Flea market.

Comparison between households with no child hunger vs. child hunger: binary variables (cross-tabs with χ^2^ statistic) and continuous variables (Student’s *t*-test). Statistical significance: ^*^p < 0.05, ^**^p < 0.01, ^***^p < 0.001.

Table 2 describes community resources such as grocery purchase, nutrition assistance program participation, and the local food environment. A greater percentage of households with child hunger shopped for groceries at a supercenter or dollar store compared to households without child hunger. La Feria area residents were more likely to shop at a supermarket for groceries (78% vs. 45.7% for Progreso, *p* <0.001); Progreso residents were more likely to shop at a supercenter (76.2% vs. 39.7%, *p* <0.001) or dollar store (43.4% vs. 1.4%, *p* <0.001). More than 63% of households received SNAP benefits. For households with child hunger, SNAP benefits did not last as long as in households without hungry children. The length of time SNAP benefits lasted was not associated with household composition (results not shown). Although SNAP participation rates were similar between the two geographic areas (65.4% for La Feria and 61.7% for Progreso), Progreso SNAP participants, compared with La Feria SNAP participants, received lower benefits each month ($334.38 vs. $382.96, *p* = 0.035), and their benefits lasted fewer days (19.7 days vs. 22.1 days, *p* = 0.008). Almost 15% of Progreso SNAP participants reported that their benefits did not last two weeks, compared with 5.1% of La Feria SNAP participants.

Almost 30% of households with children under the age of 5 years did not participate in WIC. A smaller percentage of households with child hunger reported participation in school-based nutrition programs (SBP or NSLP) or used alternative food sources (neighbor, MFV, or *pulga*), while a much larger percentage were unable to give their child or children a balanced meal during the school year or summer months. Progreso residents with school-age children reported significantly lower participation rates for both SBP and NSLP than did La Feria residents with school-age children.

The results from the RE logistic regression model (Table 3) indicated that individual, economic, and family resources were associated with child hunger; namely, lack of full-time employment and increased household size were associated with greater odds for child hunger, while having a child age 5 years reduced the odds by 56% for child hunger. Among community resources, participation in SNAP and buying food from a neighbor or friend were associated with significantly reduced odds for child hunger by 53% and 49%, respectively, while participation in NSLP was associated with increased odds for child hunger. The estimated residual intra-class correlation of the latent responses in the RE model is 0.50, indicating that 50% of the variance in residuals is attributable to differences in geographic location [42]. A stratified analysis for SNAP participants was estimated (results not shown); in addition to increased odds for child hunger in households with no adult employed full-time (OR 2.9; 95% CI 1.4, 6.0) and NSLP participation (OR 3.4; 95% CI 1.4, 8.3), the odds for child hunger were greater for households in which SNAP benefits lasted less than 22–30 days: 14 days or less (OR 2.8; 95% CI 1.3, 6.2) and 15–21 days (OR 2.4; 95% CI 1.2, 4.9).

**Table 3:** Random effects logistic regression model of childhood hunger

		**OR**
		**(95% CI)**
*Individual, economic, and family resources*		
No one employed full-time^a^		2.7^***^ (1.4, 5.0)
Household composition^b^		
2nd quartile (4)		1.3 (0.65, 2.5)
3rd quartile (5)		2.6^**^ (1.3, 5.0)
4th quartile (>5)		2.2^*^ (1.1, 4.6)
Child 5 years^c^		0.44^*^ (0.22, 0.85)
*Community resources*		
Nutrition assistance program participation		
SNAP^d^		0.47^**^ (0.28, 0.80)
NSLP^e^		4.1^***^ (1.9, 8.8)
Alternative food source		
Buy food from neighbor or friend^f^		0.51^*^ (0.29, 0.90)
Intra-class correlation (rho)	0.50^***^	
AIC	464.46	

AIC = Akaike Information Criterion.

^a^Referent: adult employed part-time or full-time.

^b^Referent: 1st quartile (2–3 adults and children residing in the household).

^c^Referent: no child 5 years of age in the household.

^d^Referent: does not participate in Supplemental Nutrition Assistant Program (SNAP).

^e^Referent: does not participate in National School Lunch Program (NSLP).

^f^Referent: does not buy food from a neighbor or friend.

Statistical significance: ^*^p < 0.05, ^**^p <0.01, ^***^p <0.001.

## Discussion

Hunger among children is a serious challenge to children’s optimal development, health, behavior, and academic performance [1,16,19,43,44]. Although child hunger is a problem faced by all children in the United States today [45], it is of increasing concern among the growing population of Mexican-origin children. This is apparently the first study to document the high prevalence of child hunger among a large sample of low-income Mexican-origin and Mexican immigrant families in Texas border *colonias*. Findings from this study focus our attention on child hunger, the most severe subcategory of food insecurity [46,47], and the presence or absence of individual, economic, family, and community resources that are associated with childhood hunger. This study relies on a comprehensive home- and community-based nutrition assessment of 470 low-income Mexican-origin families in *colonias* situated in two small towns along the Texas-Mexico border and helps answer the question: why do some very low-income families experience child hunger while others do not?

Results document the unacceptably high prevalence of child hunger and the large number of children potentially affected. Fifty-one percent of households reported child hunger, which accounted for 53% (*n* = 598) of all children. This far exceeds 2011 national estimates that 17.4% of Hispanic households had food-insecure children and 1.9% with very low food security among children [10]. It also exceeds other prior reports of Latino children of immigrant parents [6], and challenges the belief that hunger is relatively uncommon in this population [21], suggesting to us not only that child hunger should be assessed in a broader array of Latino communities rather than relying on nationwide estimates to assess its prevalence, but that national estimates do not capture the prevalence of hunger among concealed or hard-to-reach communities suffering from great poverty. Child hunger can be thought of as a serious outcome of food insecurity, constrained dietary options, and compensatory strategies [48]. Among children, hunger is a serious risk factor for long-term poor health, higher rates of chronic illness, stressful life events, poor educational achievement, and poor financial attainment in adulthood [14,19,21]. A child’s repeated exposure to hunger can be considered toxic [21].

This study not only emphasizes the alarming rates of child hunger for this sample of Mexican-origin families, but describes associations between population characteristics and the presence of child hunger. Coleman-Jensen reported that risk factors for higher rates of very low food security among children include households with children headed by a single woman, Hispanic households, and low-income households [10]. In this study of Mexican-origin households, unadjusted analyses revealed that mother’s age, education, country of birth (Mexican immigrant), household size, and household income were associated with child hunger. A greater proportion of households with child hunger relied on a friend or neighbor for transportation in order to purchase groceries, did not participate in school-based nutrition programs, or did not purchase food from a neighbor or at a *pulga*.

It is well recognized that consuming a breakfast meal is important to the nutritional health of all children and adolescents [49-51]. For low-income families, the School Breakfast Program (SBP) is an important component of the safety net for children and has been linked to improved nutrient intake [52,53]. In this sample, 38.2% of all households and 49.2% of child hunger households did not participate in SBP. In this sample, participation may be equivalent to utilization. Poor participation or utilization of SBP could result from types of foods offered or student arrival times. In areas where most children take the school bus, bus arrival may not coincide with SBP times. Underutilization of SBP could result in reduced nutrient intakes [54], and lower quality diets. Interestingly, a national study found that SBP appeared to offset food-related concerns among at-risk families, but not necessarily alleviate food insecurity once the threshold had been crossed [52]. This may help explain why 50.8% of child hunger households were SBP participants. Although the National School Lunch Program (NSLP) is the largest child nutrition assistance program [55], 37.9% of households did not participate in NSLP. Lower participation or utilization could result from available food choices or limited amount of time for lunch. Similar to SBP, 51.3% of child hunger households participated in NSLP. In the present study, a greater percentage of households that reported no adult or child hunger participated in the SBP or NSLP compared to those households with adult or child hunger. There are unknown aspects of SBP and NSLP which may contribute to the differential participation rates seen in this analysis.

Key findings from our adjusted random effects regression model document that no one in the household employed full time, household composition, and participation in the NSLP were associated with increased odds for the presence of child hunger. Lack of full-time employment may entail a limited capacity to acquire economic resources. Household composition indicates a greater demand on household food supplies operationalized through increased size of household and greater food requirements [30]. It was apparent that while participation in NSLP served to buffer some households from hunger among children, it did not do the same for all households. Although there can still be benefits from NSLP [11], for many households it may not be enough to prevent hunger among children. To address hunger among children, there are three issues that will need to be addressed: 1) difference between participation and utilization of NSLP and SBP; 2) potential need for supplemental nutrition programs for school-age children for evening meals and meals on weekends; and 3) further consideration of the adequacy of the Summer Food Service Program which is intended to substitute for the summertime absence of NSLP and SBP. Interestingly, households with a child 5 years, which suggests ineligibility for WIC or SBP/NSLP for that child, were 56% less likely report hunger among children. This may be explained by higher participation rates of these households in SNAP.

There were two types of community resources that were protective: participation in SNAP and purchasing food from a neighbor or friend were associated with reduced odds for child hunger. Contrary to prior work with Latino immigrant children that found no association between food stamps (SNAP) and child hunger [6], this study found, independent of economic and family resources that SNAP-participating households were more than two times less likely to report child hunger than households that did not participate in SNAP. SNAP, which is the largest food assistance program in the U.S. [55], is known to free up household resources [44], reduce very low food security over time [56], and minimize the positive associations of household and child food insecurity with children’s poor health [57]. Still, SNAP participation does not prevent hunger or food insecurity [4,58]. In this sample, 59% of households with child hunger were SNAP recipients. This calls into question the adequacy of SNAP benefits, which is influenced by financial and time resources and individual, household, and community factors that impact the purchasing power of benefits [59]. Although this is a common finding among cross sectional analyses of SNAP and food insecurity, Nord and colleagues discovered in a longitudinal analysis that positive associations are the result of self-selection into SNAP by families as they first go without food, and that SNAP eventually plays a palliative role for household hunger [56]. The negative association between SNAP and child hunger in this study is, we believe, indicative of households in receipt of SNAP benefits that commonly experience food insecurity and have thus largely moved beyond a tipping point for self-selection into SNAP participation. Although participation in SNAP was protective from child hunger in this sample, child hunger was present in 59% of households utilizing SNAP, and SNAP was not enough to prevent child hunger. This is more than twice the prevalence rate (23%) among low-income families previously reported in CCHIP [4]. In part, this child hunger may result from the level of support, household size, length of time that benefits last, budget management skills, or amount of competing demands for limited resources. In our subgroup analysis of SNAP participants, the odds for childhood hunger were greater for households in which SNAP benefits lasted less than all month. In addition, a large percentage of participants were not utilizing SNAP, perhaps the result of perceived or actual eligibility for individual household members (e.g., mixed-status families with an undocumented parent and U.S.-born child), [60] language or educational barriers, immigration status and fear of deportation, or fear of stigmatization [47,61,62]. A recent report from the Institute of Medicine made several recommendations to increase the adequacy of SNAP benefits [59]. These include consideration of specific individual, household, and environmental factors on determining the adequacy of SNAP allotment, which recognizes cost–time trade-offs, geographic variation in food prices, and spatial access to retail food sources.

There has been little consideration given to alternative food sources, which represent a unique and previously unmeasured compensatory strategy to improve food security [34]. In this study, the odds of child hunger in households whose inhabitants purchased food from a neighbor or friend were 49% less likely than households that did not rely on this compensatory practice. This strategy illustrates resiliency and community connectedness and may provide *colonia* residents with the opportunity to preserve limited economic and family resources by reducing travel cost and increasing the frequency at which food may be purchased as needed and in smaller quantities.

There are several limitations to this cross-sectional study. First, immigration status (e.g., naturalized citizen of the U.S. or legal resident) was not assessed [47]. Thus, we do not know what percentage of the 322 Mexican immigrants in this study were undocumented or were in mixed-status households. This may explain why a significantly larger percentage of Mexican immigrant households reported hungry children. Prior studies reported that limited access to safety net public assistance may contribute to high levels of hunger, especially for the undocumented, but could also affect legal immigrants [47]. In other studies of Latino immigrants, immigration status was not assessed [14,46,63]. Second, adult and child experiences of hunger were based only on mothers’ report. Prior work demonstrated that children are able to report their own food insecurity experiences, which may differ from proxy reports by the mother [12,64]. Third, data were not available regarding forms of material hardship unrelated to food insecurity that result in competing demands for strained household economic resources. Fourth, the data did not attribute child hunger to specific children, or how the availability of food varied among household members. Fifth, data were not available on the frequency or duration of adult or child hunger. Was this persistent or new? In a recent government report, of households that reported child(ren) were hungry, 23% experienced the condition almost every month and 51% endured it for a period of several months [65]. Finally, there is some concern on the low reporting of participation in SBP and NSLP, which may be indicative of lack of utilization of the programs or not knowing the names of the programs within the schools.

## Conclusions

Notwithstanding these limitations, there are a number of strengths to this study, including a large sample of immigrant and hard-to-reach Mexican-origin households and comprehensive data collected in Spanish during in-person interviews conducted by trained *promotora*-researchers. The results of this study further our understanding of child hunger among Mexican-origin households in Texas border *colonias*. In documenting an unacceptably high exposure of children to hunger, which may influence dietary intake and health outcomes, the results confirm the important role of safety net programs, such as SNAP. However, the results may understate the degree of child hunger during the summer months when children are not in school and away from SBP and NSLP [11]. A large percentage of potentially eligible households did not participate in these nutrition assistance programs. There may be restrictions on immigrants which may ultimately affect the health of children. Efforts must be made to increase access to food-related resources, expand the availability of meals for low-income children, and ensure that all eligible families, especially mixed-status families receive nutrition assistance. In the end, child hunger raises serious concerns for communities. Considering the important relationship between child hunger and adverse mental and physical health outcomes among low-income children [19], this study underscores the importance of identifying the presence of child hunger among low-income Mexican-origin children and increasing access to nutrition-related resources. Health inequities at younger ages among the Mexican-origin population are likely to persist across the lifespan and into old age, with an accumulation of risk [66]. Our findings support the identification of child hunger as a possible target for screening and interventions to prevent poor developmental and health outcomes, and the importance of improved culturally-specific communication with Mexican-origin residents about availability of community and federal nutrition resources [61]. SNAP and school-based nutrition assistance programs are critical to millions of people, especially children. However, in the case of SNAP, efforts should be undertaken as suggested by the recent IOM report to define the adequacy of the SNAP allotment and make adjustments accordingly [59]. In the case of school-based nutrition programs, acceptable meals and sufficient time for consuming SBP and NSLP meals should be made available to all children at risk of or experiencing hunger. Creative approaches, such as provision of a third meal and meals on weekends, should be explored. Our greatest return on the investment in these feeding programs is in ensuring that all children and families have access to affordable and healthy food supplies. Further explorations on strategies to address child hunger with young mothers are needed that incorporate both knowledge and skill-building. Ideally, this would include the empowerment of *promotoras* to deliver culturally- and linguistically-appropriate interventions and strategies.

## Competing interests

The authors declare that they have no competing interests.

## Authors’ contributions

JRS designed the study, and worked on the development of the instrument and the protocol for collection of data. JRS, WRD, and CCN wrote the first draft of the paper. JRS, WRD, and CCN read and approved the final manuscript.

## Authors’ information

JRS is Professor of Health Promotion and Community Health Sciences and Founding Director of the Program for Research and Outreach-Engagement on Nutrition and Health Disparities; CCN is a former Program Coordinator and Data Manager; and WRD is Assistant Professor of Health Promotion and Community Health Sciences.

## Pre-publication history

The pre-publication history for this paper can be accessed here:

http://www.biomedcentral.com/1471-2431/13/143/prepub

## References

[B1] Ludwig DS Blumenthal SJ Willett WC Opportunities to reduce childhood hunger and obesity JAMA 2012 308 24 2567 2568 23268513 10.1001/jama.2012.45420

[B2] Swaminathan MS Combating hunger Science 2012 338 1009 23180830 10.1126/science.1231305

[B3] Wehler CA Scott RI Anderson JJ The community childhood hunger identification project: a model of domestic huner - demonstration project in Seattle, Washington J Nutr Educ 1992 24 29S 35S

[B4] Lewit EM Kerrebrock N Childhood hunger Future Child 1997 7 1 128 137 9170740

[B5] Nord M Food insecurity in households with children: prevalence, severity, and household characteristics 2009 Economic Research Service: United States Department of Agriculture

[B6] Kersey M Geppert J Cutts DB Hunger in young children of Mexican immigrant families Public Health Nutr 2007 10 4 390 395 17362535 10.1017/S1368980007334071

[B7] Treviño RP Marshall RM Hale DE Rodriguez R Baker G Gomez J Diabetes risk factors in low-income Mexican-American children Diabetes Care 1999 22 202 207 10333934 10.2337/diacare.22.2.202

[B8] Ogden CL Carroll MD Curtin LR Lamb MM Flegal KM Prevalence of high body mass index in US children and adolescents, 2007–2008 JAMA 2010 303 3 242 249 20071470 10.1001/jama.2009.2012

[B9] Wang Y Beydoun MA The obesity epidemic in the United States – gender, age, socioeconomic, racial/ethnic, and geographic characteristics: a systematic review and meta-regression analysis Epidemiol Rev 2007 29 6 28 17510091 10.1093/epirev/mxm007

[B10] Coleman-Jensen A Nord M Andrews M Carlson S Household food security in the United States in 2011 2012 Washington, DC: Economic Research Service, USDA

[B11] Nord M Romig K Hunger in the summer: seasonal food insecurity and the national school lunch and summer food service programs J Child Poverty 2006 12 2 141 158

[B12] Sharkey JR Nalty C Johnson CM Dean WR Children’s very low food security is associated with increased dietary intakes in energy, fat, and added sugar among Mexican-origin children (6–11 y) in Texas border *colonias* BMC Pediatr 2012 12 16 22348599 10.1186/1471-2431-12-16 PMC3298490

[B13] Cutts DB Pheley AM Geppert JS Hunger in midwestern inner-city young children Arch Pediatr Adolesc Med 1998 152 489 493 9605034 10.1001/archpedi.152.5.489

[B14] Chilton M Black MM Berkowitz C Casey PH Cook J Cutts D Jacobs RR Heeren T Cuba SE Coleman S Food insecurity and risk of poor health among US-born children of immigrants Am J Public Health 2009 99 556 562 19106417 10.2105/AJPH.2008.144394 PMC2661461

[B15] Center on Hunger and Poverty The consequences of hunger and food insecurity for children 2002 Waltham: MA: Brandeis University

[B16] Alaimo K Olson CM Frongillo EA Food insufficiency and American school-aged children’s cognitive, academic, and psychosocial development Pediatrics 2001 108 1 44 53 11433053

[B17] Slopen N Fitzmaurice G Williams DR Gilman SE Poverty, food insecurity, and the behavior for childhood internalizing and externalizing disorders J Am Acad Child Adolesc Psychiatry 2010 49 5 444 452 20431464 10.1097/00004583-201005000-00005

[B18] Kursmark M Weitzman M Recent findings concerning childhood food security Curr Opin Clin Nutr Metab Care 2009 12 3 310 316 19333121 10.1097/MCO.0b013e3283298e37

[B19] Weinreb L Wehler C Perloff J Scott R Hosmer D Sagor L Gundersen C Hunger: its impact on children’s health and mental health Pediatrics 2002 110 4 e41 12359814 10.1542/peds.110.4.e41

[B20] Research F Center A Community childhood hunger idenitifcation project: a survey of childhood hunger in the United States 1995 Washington, DC: FRAC

[B21] Kirkpatrick SI McIntyre L Potestio ML Child hunger and long-term adverse consequences for health Arch Pediatr Adolesc Med 2010 164 8 754 762 20679167 10.1001/archpediatrics.2010.117

[B22] Whitaker RC Phillips SM Orzol SM Food insecurity and the risks of depression and anxiety in mothers and behavior problems in their preschool-age children Pediatrics 2006 118 3 e859 e868 16950971 10.1542/peds.2006-0239

[B23] Murphy JM Wehler CA Pagano ME Little M Kleinman RE Jellinek MS Relationship between hunger and psychosocial functioning in low-income American children J Am Acad Child Adolesc Psychiatry 1998 37 2 163 170 9473912 10.1097/00004583-199802000-00008

[B24] Cook JT Frank DA Food security, poverty, and human development in the United States Ann NY Acad Sci 2008 1136 193 209 17954670 10.1196/annals.1425.001

[B25] Alaimo K Olson CM Frongillo EA Briefel RR Food insufficiency, family income, and health in US preschool and school-aged children Am J Public Health 2001 91 781 786 11344887 10.2105/ajph.91.5.781 PMC1446676

[B26] Quandt SA Shoaf JI Tapia J Hernández-Pelletier M Clark HM Arcury TA Experiences of Latino immigrant families in North Carolina help explain elevated levels of food insecurity and hunger J Nutr 2006 136 10 2638 2644 16988139 10.1093/jn/136.10.2638 PMC1626531

[B27] Sharkey JR Dean WR Johnson CM Association of household and community characteristics with adult and child food insecurity among Mexican-origin households in *colonias* along the Texas-Mexico border Int J Equity Health 2011 10 19 21569496 10.1186/1475-9276-10-19 PMC3118344

[B28] Migration information source: Mexican immigrants in the United Stateshttp://www.migrationinformation.org

[B29] Feeding America Map the Meal Gap: Map the Meal Gap: Child Food Insecurity 2012 2013 Chicago, IL: Feeding America http://feedingamerica.org/hunger-in-america/hunger-studies/map-the-meal-gap/~/media/Files/a-map-2011/2011-mmg-exec-summary.aspx#

[B30] Wehler C Weinreb LF Huntington N Scott R Hosmer D Fletcher K Goldberg R Gundersen C Risk and protective factors for adult and child hunger among low-income housed and homeless female-headed families Am J Public Health 2004 94 109 115 14713707 10.2105/ajph.94.1.109 PMC1449835

[B31] Esparza AX Donelson AJ The colonias reader: economy, housing and public health in U.S.-Mexico 2010 Tucson, AZ: The University of Arizona Press

[B32] Sharkey JR Horel S Han D Huber JC Association between Neighborhood Need and Spatial Access to Food Stores and Fast Food Restaurants in Neighborhoods of colonias Int J Health Geogr 2009 8 9 19220879 10.1186/1476-072X-8-9 PMC2653484

[B33] Johnson CM Sharkey JR Dean WR John JAS Castillo MD *Promotoras* as research partners to engage health disparity communities J Acad Nutr Diet 2013 113 5 638 642 23375463 10.1016/j.jand.2012.11.014 PMC3633728

[B34] Sharkey JR Dean WR Johnson CM Use of *Vendedores* (Mobile food vendors), *Pulgas* (Flea markets), and *Vecinos o Amigos* (Neighbors or friends) as alternative sources of food for purchase among Mexican-origin households in Texas border *colonias* J Acad Nutr Diet 2012 112 705 710 22709775 10.1016/j.jand.2011.12.006 PMC3378981

[B35] Dean WR Sharkey JR John JS Pulga (Flea market) contributions to the retail food environment of colonias in the South Texas border region J Am Diet Assoc 2011 111 705 710 21515116 10.1016/j.jada.2011.02.009 PMC3082738

[B36] Kendall A Olson CM Frongillo EA Validation of the Radimer/Cornell measures of hunger and food insecurity J Nutr 1995 125 2793 2801 7472659 10.1093/jn/125.11.2793

[B37] Radimer K Olson C Campbell C Development of indicators to assess hunger J Nutr 1990 120 11 Suppl 1544 1548 2243303 10.1093/jn/120.suppl_11.1544

[B38] Goldstein H Multilevel statistical models 2011 4 New York: John Wiley & Sons

[B39] Kleinbaum DG Kupper LW Muller KE Nizam A Applied regression analysis and other multivariate models 1998 3 Pacific Grove: Duxbury Press

[B40] Akaike H A new look at the statistical model identification IEEE Trans Autom Control 1974 19 6 716 723

[B41] Akaike H Likelihood and the Bayes procedure Trab Estad Investig Oper 1980 31 1 143 166

[B42] Rabe-Hesketh S Skrondal A Measures of dependence and heterogeneity Multilevel and longitudinal modeling using stata volume II 2012 College Station: Stata Press 532 534

[B43] Jyoti DF Frongillo EA Jones SJ Food security affects school children’s academic performance, weight gain, and social skills J Nutr 2005 135 2831 2839 16317128 10.1093/jn/135.12.2831

[B44] Gundersen C Kreider B Bounding the effects of food insecurity on children’s health outcomes J Health Econ 2009 28 971 983 19631399 10.1016/j.jhealeco.2009.06.012

[B45] Gundersen CG Garasky SB Financial management skills are associated with food insecurity in a sample of households with children in the United States J Nutr 2012 142 1865 1870 22955515 10.3945/jn.112.162214

[B46] Himmelgreen DA Pérez-Escamilla R Segura-Millán S Peng Y-K Gonzalez A Singer M Ferris A Food insecurity among low-income Hispanics in Hartford, Connecticut: implications for public health policy Hum Organ 2000 59 3 334 342

[B47] Hadley C Galea S Nandi V Nandi A Lopez G Strongarone S Ompad D Hunger and health among undocumented Mexican immigrants in a US urban area Public Health Nutr 2007 11 2 151 158 17610762 10.1017/S1368980007000407

[B48] Seligman HK Schollinger D Hunger and socioeconomic disparities in chronic disease N Engl J Med 2010 363 1 6 9 20592297 10.1056/NEJMp1000072

[B49] Affenito SG Breakfast: a missed opportunity J Am Diet Assoc 2007 107 4 565 569 17383260 10.1016/j.jada.2007.01.011

[B50] Affenito SG Thompson DR Barton BA Franko DL Daniels SR Obarzanek E Schreiber GB Striegel-Moore RH Breakfast consumption by African-American and white adolescent girls correlates positively with calcium and fiber intake and negatively with body mass index J Am Diet Assoc 2005 105 938 945 15942545 10.1016/j.jada.2005.03.003

[B51] Chitra U Reddy CR The role of breakfast in nutrient intake of urban schoolchildren Public Health Nutr 2007 10 1 55 58 17212843 10.1017/S1368980007219640

[B52] Bartfeld JS Ahn H-M The school breakfast program strengthens household food security among low-income households with elementary school children J Nutr 2011 141 470 475 21228262 10.3945/jn.110.130823

[B53] Affenito SG Thompson D Dorazio A Albertson AM Loew A Holschuh NM Ready-to-eat cereal consumption and the school breakfast program: relationship to nutrient intake and weight J Sch Health 2013 83 28 35 23253288 10.1111/j.1746-1561.2012.00744.x

[B54] Kleinman RE Hall S Green H Korzec-Ramirez D Patton K Pagano ME Murphy JM Diet, breakfast, and academic performance in children Ann Nutr Metab 2002 46 Suppl 1 24 30 12428078 10.1159/000066399 PMC3275817

[B55] American Dietetic Association Position of the American dietetic association: child and adolescent nutrition assistance programs J Am Diet Assoc 2010 110 5 791 799 20440910 10.1016/j.jada.2010.02.025

[B56] Nord M Golla AM Does SNAP decrease food insecurity? untangling the self-selection effect Economic research report: volume report No. 85 2009 Washington: U.S. Dept. of Agriculture, Economic Research Service

[B57] Cook JT Frank DA Levenson SM Neault NB Heeren TC Black MM Berkowitz C Casey PH Meyers AF Cutts DB Child food insecurity increases risks posed by household food insecurity to young children’s health J Nutr 2006 136 1073 1076 16549481 10.1093/jn/136.4.1073

[B58] Dhokarh R Himmelgreen DA Peng Y-K Segura-Pérez S Hromi-Fiedler A Pérez-Excamilla R Food insecurity is associated with acculturation and social networks in Puerto Rican households J Nutr Educ Behav 2011 43 288 294 20952259 10.1016/j.jneb.2009.11.004 PMC3023827

[B59] Caswell JA Yaktine AL Supplemental nutrition assistance program: examining the evidence to define benefit adequacy 2013 Washington: National Academy of Sciences 24901188

[B60] Xu Q Brabeck K Service utilization for Latino children in mixed-status families Soc Work Res 2012 36 3 209 221

[B61] Rivera-Ottenberger A Werby E Latino participation in food assistance programs: a study conducted for project bread Volume Paper 13 2007 Boston: Center for Social Policy Publications, University of Massachusetts Boston

[B62] Geltman PL Meyers AF Immigration legal status and use of public programs and prenatal care J Immigr Health 1999 1 2 91 97 16228707 10.1023/A:1021832422075

[B63] Kaiser LL Legar-Quiñonez HR Lamp CL Johns MC Sutherlin JM Harwood JO Food security and nutritional outcomes of preschool-age Mexican-American children J Am Diet Assoc 2002 102 924 929 12146552 10.1016/s0002-8223(02)90210-5

[B64] Nalty CC Sharkey JR Dean WR Children’s reporting of food insecurity in predominately food insecure households in Texas border colonias Nutr J 2013 12 15 23356877 10.1186/1475-2891-12-15 PMC3598463

[B65] Coleman-Jensen A Mark N Margaret A Steven C Statistical Supplement to Household Food Security in the United States in 2010, AP-057. U.S. Dept. of Agriculture Econ Res Serv 2011

[B66] Villa VM Wallace SP Bagdasaryan S Aranda MP Hispanic baby boomers: health inequities likely to persist in old age Gerontologist 2012 52 2 166 176 22399578 10.1093/geront/gns002 PMC3304894

